# The Association of COVID-19 Stressors and Family Health on Overeating before and during the COVID-19 Pandemic

**DOI:** 10.3390/ijerph19106174

**Published:** 2022-05-19

**Authors:** Rahee Kim, Eliza Olpin, Lynneth Kirsten Novilla, AliceAnn Crandall

**Affiliations:** Department of Public Health, Brigham Young University, Provo, UT 84602, USA; raheekim1029@gmail.com (R.K.); eliza@brharding.net (E.O.); kirsten.novilla@gmail.com (L.K.N.)

**Keywords:** overeating, family health, COVID-19 stressors, family, Mturk

## Abstract

Prior studies have examined how stress and the family environment affect overeating, but less is known about how COVID-19 stressors and family health may affect overeating during the COVID-19 pandemic. The research questions included: (1) Did COVID-19-related stressors increase the risk for overeating among adults in the United States? (2) Did family health protect against overeating during a pandemic? The sample included 443 participants aged 18 years and older living in the United States who were recruited via Amazon Mechanical Turk. Stata version 16 was used to analyze the data using multiple linear regression. The results indicate that one year into the pandemic, COVID-19 stressors were associated with increased overeating, even after adjusting for overeating before the pandemic. More family health resources were associated with less overeating. These results indicate that although COVID-19 stressors were associated with overeating, greater family health resources helped prevent overeating. Interventions and policies that aim to increase health resources for families may be particularly beneficial at preventing overeating and obesity in the face of long- and short-term stress.

## 1. Introduction

### 1.1. The Impact of COVID-19 on Overeating

COVID-19 has impacted several facets of life for individuals and families as the circumstances surrounding the pandemic have posed significant challenges to mental health, social relationships, and economic well-being [1,2,3]. Although COVID-19 is a novel stressful event, the negative effects of stressful changes on mental health and emotional well-being are well understood. For example, past studies have indicated that experiencing loneliness can trigger or increase the risk of overeating [4] because of the sense of relief and comfort that eating can provide [5,6,7,8,9,10]. Overeating is characterized as eating large amounts of food or consuming more calories than are used in a day [11].

Given the multi-faceted role of COVID-19 in inducing several stressors, including uncertainty, fear, social isolation, lack of physical activity, nutritional imbalances [12], and unemployment or financial uncertainty [13], it is not surprising that COVID-19 has created a domino effect for increasing levels of anxiety, depression, and unhealthy coping behaviors such as overeating [14,15]. Recent research indicates that the COVID-19 lockdowns have contributed to negative dietary changes due to the length of time of the lockdowns, as the lack of varied social interactions have decreased and taken a toll on mental and emotional health [16,17,18]. Studies in the U.S. and U.K. have found increased levels of loneliness and poorer coping mechanisms among adults since the pandemic began [19,20]. Increased loneliness during the lockdown led to feelings of stress, emptiness, and boredom, which contributed to the increased consumption of foods, especially unhealthy foods [21]. This finding was supported by a study conducted in Australia during the COVID-19 lockdown. Owen et al. (2021) discovered that those who felt more stress and isolation during this stressful period were more likely to report poor appetite or overeating [22].

### 1.2. Why Family Health Would Affect Overeating

During the COVID-19 pandemic, many individuals found themselves spending more time at home due to local shutdowns. Furthermore, stressors such as economic uncertainty, school closures, limited supplies, fear, uncertainty, and social isolation were felt by many households and extended families [23,24,25]. Families have an impact on dietary behaviors, and the increased time with families could have positive or negative effects on eating. In a study of food and eating as social practice, researchers emphasized that “families are created through relationships involving food and that feeding a family is an activity central to family life” [26]. Another study examining the relationship between parent and child eating behaviors indicated that the way children eat is strongly predicted by the mother’s food intake and mirrors primary caregivers’ eating style and food intake [27]. Although most research in this area is child-centered, a few studies suggest that even in adulthood, eating behaviors can be susceptible to family influence. For example, marital transitions or moving in with a partner have been linked to changes in diet [28,29,30]. These changes are likely due in part to new environmental and relationship stressors. A study of 300 college students found that family variables could account for more than 18% of the variance in compulsive eating behaviors among males. Males who tended to eat compulsively reported feeling dissatisfied with parental relationships and perceived their family as “incohesive and rigid” [31].

In recent years, researchers studying the family system and environment in the context of public health have used the following definition of family health: “A resource at the level of the family unit that develops from the intersection of the health of each family member, their interactions and capacities, as well as the family’s physical, social, emotional, economic, and medical resources” [32]. Family health includes: (1) a family’s internal and external health resources, such as access to transportation to get help, trust in healthcare workers, adequate housing, and financial security; (2) family external social supports; (3) the family’s social and emotional health processes, such as good communication and feelings of belonging; and (4) the family’s habits and culture regarding a healthy lifestyle [33].

Family health resources include both internal and material resources, such as the effects of family member health on family routines, trust in medical professionals, knowledge of outside resources, health insurance coverage, access to transportation, adequate housing, and so forth [33]. These resources may be particularly important to individual eating behaviors. Income, reliable access to food, and time availability are all resources that, when insufficient, may be associated with overeating [34,35,36]. Several researchers have found that household food insecurity negatively affects overeating and obesity in both adults and children [37,38,39,40]. In addition, individual member mental health is an internal family health resource. Research has shown an association between depressive symptoms and emotional eating, as well as higher consumption of sweet foods [41,42]. Other family health resources include the ability to manage stress and work–family conflict. Some studies have shown that work–family conflict can lead to coping mechanisms that interfere with a healthy diet, such as emotional eating and choosing high-fat foods [43,44,45].

### 1.3. Demographics and Overeating

The extant literature indicates that some sociodemographic characteristics, such as gender, race, and marital status, may affect the tendency to overeat. For example, a study of the gender differences in eating disorder symptoms found that while women were more likely to feel a loss of control when eating, men were more likely to report overeating [46]. However, other studies concluded that females are more likely to overeat when stressed than males [47,48]. In a study of the effects of COVID-19 on overeating and loss of appetite, Australian researchers found that individuals living with a partner and without children were the least likely to experience loss of appetite or overeat, compared with individuals living alone, single parents, or people sharing housing with non-family members [22]. When it comes to age and overeating, little is known about overeating in middle adulthood. Most studies have focused on groups that fall in the adolescent to early adulthood years age bracket, as it is the age where overeating is most likely to occur [48].

### 1.4. Aims/Purpose

Although the extant literature, mostly relating to children and adolescents, indicates a relationship between stress and overeating, and the importance of the family environment on overeating, less is known about how COVID-19 stressors and the health of the family may affect overeating during the COVID-19 pandemic, particularly among adults. To address this gap, the current study includes the following two research questions: (1) Did COVID-19-related stressors increase the risk for overeating among adults in the United States? We hypothesized that COVID-19-related stressors would lead to worse overeating symptoms. (2) Did better family health reduce overeating during a pandemic? We hypothesized that better family health would be associated with less overeating, and that family health resources, in particular, would be protective against overeating during the pandemic.

## 2. Materials and Methods

The study was conducted in March 2021, one year after the COVID-19 pandemic began in the United States. Participants included 443 individuals ages 18 and older living in the United States who were workers on Amazon Mechanical Turk (MTurk). Based on their MTurk profile, registered MTurk workers who met the qualifications (at least 18 years of age and living in the United States) could access the study. Participants completed a 15-min survey on Qualtrics. After completing the survey, participants received a USD 2.00 reward credited to their MTurk account. The study was approved by the Brigham Young University institutional review board.

### 2.1. Measures

#### 2.1.1. Overeating

Overeating was measured using the COVID-19 Isolation Eating Scale (CIES) [49]. This 10-item questionnaire assesses eating behaviors before and during confinement, using eating disorder symptoms according to the DSM-5 criteria for binge eating disorders [50]. The scale was modified for a non-clinical population so that participants could report their overeating symptoms before and during the COVID-19 pandemic. Response options were on a 5-point Likert Scale ranging from (1) never to (5) always before the COVID-19 pandemic and currently. The responses were totaled across the ten items for a total score for overeating before COVID-19 (included as a control) and overeating currently (dependent variable). Scores could range from 10 to 50. Sample items included ‘Eat when negative emotions are experienced such as fear, anxiety, sadness, loneliness, anger or boredom’ and ‘Eat constantly throughout the day without realizing what is happening.’ Cronbach’s alpha from prior studies ranged from good (α = 0.81) to excellent (α = 0.92) [49]. The current sample also had high internal reliability with a Cronbach’s alpha of 0.95.

#### 2.1.2. COVID-19 Stressors

Three COVID-19 stressors were included: whether participants had had COVID-19; whether they had moved due to COVID-19; and whether their employment or income had been affected because of COVID-19. Employment/income effects included losing a job, having reduced hours or income, and/or having to take on an additional job to make ends meet. The three stressors were summed for a total COVID-19 stressors score ranging from 0 to 3 points.

#### 2.1.3. Family Health

Family health was measured using the Family Health Scale (FHS) [33]. For the purposes of this study, the nine-item family health resources subscale was included as it has been found to be highly associated with health outcomes during COVID-19 [51]. Along with family health resources, the Family Health Scale-Short Form (FHS-SF) that measures a unidimensional construct of family health was included. The three items relating to family health resources were excluded from the short-form measure. The remaining seven items in the FHS-SF covered the family’s general health based on social and emotional health processes, external social supports, and healthy behaviors. Response options for each of the FHS items were on a 5-point Likert Scale ranging from (1) strongly disagree to (5) strongly agree. Negatively worded items were reverse coded so that higher scores indicated better family health. Sample items from the FHS-SF included ‘In my family we support each other’ and ‘In my family I feel safe in my family relationships.’ Sample family health resources items included ‘In my family we do not trust doctors and other health professionals’ and ‘In the past 30 days family worries and problems distracted me when I was working.’ Previous studies have found good internal reliability for the FHS (family health resources α = 0.82; 10-item FHS-SF α = 0.84) [33]. The current sample also had strong internal reliability (family health resources: α = 0.93; 7-item FHS-SF: α = 0.84).

#### 2.1.4. Controls

The following controls were included in the final model: participant gender (1 = female; 0 = male), age in years, marital status (1 = married or cohabitating; 0 = not married/cohabitating), education (1 = bachelor’s degree or higher, 0 = less than a bachelor’s degree), race (1 = White; 0 = non-White) and overeating before the pandemic.

## 3. Data Analysis

Data were cleaned and analyzed in Stata version 16 (StataCorp LLC, College Station, TX, USA). Multiple linear regression was conducted to examine the effects of family health and COVID-19 stressors on overeating behaviors one year into the pandemic. Overeating behaviors prior to the pandemic, age, sex, marital status, race, and education were included as controls in the final models.

## 4. Results

The mean age of participants was 37 years old, 38% were female, 65% were cohabitated or married, 74% had a bachelor’s degree or higher, and 71% reported their race as White. Participants had on average 1.3 COVID-19-related stressors. Mean scores for overeating were 14.72 before the COVID-19 pandemic; the mean score of overeating was 15.51 one year after the COVID-19 pandemic began (see Table 1 for the full descriptive data for the sample).

In the final model (Table 2), COVID-19 stressors were associated with increased overeating one year into the pandemic (0.63, *p* < 0.05), after controlling for prior overeating. General family health was not associated with overeating during the COVID-19 pandemic, but higher family health resources were associated with decreased overeating one year into the pandemic (−1.03, *p* < 0.001).

## 5. Discussion

Consistent with our first hypothesis, COVID-19 stressors were associated with more overeating, even after controlling for overeating prior to the pandemic. Consistent with hypothesis (2), family health resources were associated with less overeating since the start of the COVID-19 pandemic. However, other aspects of family health did not contribute to overeating.

### 5.1. COVID-19 and Overeating

Aligned with the existing literature demonstrating that stressors lead to more overeating, the current study results indicate an association between COVID-19 stressors and overeating. This relationship held true even when controlling for prior overeating behaviors. This finding is of particular importance as it is indicative of the profound effect that pandemic stressors have had and may continue to have on coping behaviors such as overeating. One reason for this relationship may be that increased loneliness, uncertainty, fear, and disruptions to regular routines can lead to emotional overeating to cope with the pandemic’s circumstances [16,17,18].

Another interesting result of this study was that higher education was found to be a significant protective factor against overeating during COVID-19. This study showed almost a full point decrease in overeating during COVID-19 if the participant had a bachelor’s degree or higher. This may be because higher education means more material resources, lower stress levels, and an increased resource pool to handle stressful events, all of which can protect against using overeating as a coping mechanism [52].

In the current study, we measured specific stressors relating to COVID-19 (e.g., job loss, having COVID-19, and moving). Other studies have found that higher general stress during COVID-19, as measured using the Perceived Stress Scale, was associated with more eating between meals, higher consumption of less healthy foods, and more weight gain [53]. Thus, it is likely that stress in general during COVID-19, rather than specific stressors, led to more overeating and less healthy eating patterns. Healthy coping mechanisms such as mindfulness are thus an important intervention method for preventing overeating in stressful contexts in general.

### 5.2. Family Health and Overeating

The study results indicate that family health resources, but not other aspects of family health, were significantly associated with overeating behaviors, with a higher family health resources score being associated with less overeating. During the COVID-19 pandemic, unemployment, financial uncertainty, shortages, and adjustments to public transportation in many cities might have put additional pressure on previously food-secure households. Family health resources may have helped to prevent these challenges. Additionally, as many parents were forced to work from home during the pandemic, sometimes also juggling childcare and schooling, it is possible that the increase in family distractions from work may have led to more overeating as a coping mechanism in some households. In stressful situations, such as the COVID-19 pandemic, families often rely more heavily on external social support systems. Having someone to turn to for a loan, transportation, advice, childcare, or help when facing a problem, score higher for external social support on the family health scale. Social capital is another way of quantifying how well connected a person is to networks that may offer information or support. Like financial resources that protect against food insecurity, social capital can act as a stress buffer in families undergoing the challenges and pressures of life, which in turn may reduce unhealthy coping mechanisms. For instance, a study of 84 mother–child dyads in Montreal found that “mothers’ social capital moderated the association between maternal stress and emotional overeating” [54]. Further research with longitudinal data will help to better understand how family health may vary over time, and how these variations may affect overeating.

## 6. Limitations

The results should be considered in light of some study limitations. First, this study was a cross-sectional data analysis, which prevents drawing causal conclusions. Additionally, we were unable to assess whether family health had changed as a result of the pandemic or was static. Understanding how COVID-19 stressors may have affected different aspects of family health and the longitudinal invariance of the FHS would be useful in more fully understanding its effects on overeating. Another limitation is that the participants were surveyed 12 months after the start of the pandemic and asked to report current overeating and recall symptoms of overeating prior to the pandemic. As a result, there may be an issue of recall bias on overeating prior to the pandemic. Despite this limitation, the inclusion of prior overeating as control does help to tease out the effects of the current pandemic on overeating versus perceived chronic overeating. Another limitation of the study is that overeating was based on self-reported eating behaviors. Overeating was likely underreported because it is a less socially desired behavior. Historically, under-reporting dietary intake has been a challenge in research on dietary behaviors [55].

Additionally, although prevailing theory indicates that feelings of loneliness, uncertainty, fear, anxiety and other emotional responses were the mediating causes for overeating, we did not measure these factors. Finally, although prior studies have found that MTurk has strong generalizability to national samples [56,57], it is nevertheless a convenience sample. The results may not be generalizable to other populations in the U.S. Further research should be conducted in a representative national sample and in low-income and minority samples to assess whether the results are similar to the current study.

## 7. Implications

Focusing on understanding how disruption to regular individual and family routines may trigger overeating can also serve to help to address the growing obesity epidemic in the United States. A recent survey estimated that 73.6% of Americans are overweight or obese [58]. In addition, several studies have examined family-based healthy eating interventions with the goal of improving the nutrition outcomes of children [59,60]. Psychosocial factors, particularly family interactions regarding healthy eating, play a significant role in healthy eating patterns [61]. One of the primary factors that influence healthy eating patterns is the amount of time available for eating activities, such as time spent cooking, learning new recipes, improving cooking skills, and sharing meals with family members [62]. However, there is a lack of research about interventions targeted at adults and childless households that would also benefit from additional information regarding disordered eating behaviors in the family context.

The tendency to overeat when stressed is a common problem, yet few interventions have been developed to address this issue among adults. Existing interventions frequently use a mindfulness approach to reduce static and emotional eating. One advantage of mindfulness is that it can be easily performed at home. However, while promising, the evidence is still inconclusive regarding the long-term effectiveness of this type of intervention [63,64,65,66,67]. Additionally, the results of this study indicate that perhaps mindfulness interventions overlook an important variable in overeating, that is, family health resources. A new approach to overeating interventions focused on increasing family coping and material resources has the potential to address the root causes of overeating as a stress response. Based on the current study and prior research, this aim is best accomplished at a family level, as families play an important role in improving overeating symptoms.

Connecting families in high-stress situations to external resources is one of the first steps to protect family well-being and reduce negative coping mechanisms. At Stanford Children’s hospital, the Department of Family-Centered Care created a program to address heightened food insecurity due to the COVID-19 pandemic among patients’ families, including take-home bags, grocery deliveries, and gift cards [68]. More research is needed on other programs and policies that aim to provide resources to families, such as rental assistance programs and the Supplemental Nutrition Assistance Program, and their effect on overeating behaviors. In addition, more programs and policies are needed that go beyond consumer knowledge to influence the global supply chain—for example, reducing the availability of food products with high sugar or salt content, making them easier to overeat. Efforts should be made to make evidence-based programs more readily accessible (e.g., cost, awareness of the program, etc.) to families.

## 8. Conclusions

As COVID-19 triggered many stressors, increased feelings of loneliness, uncertainty, fear, and change to daily routines may lead to emotionally driven overeating in response to the pandemic’s conditions. The current study found that even after adjusting for overeating prior to the pandemic, there was still an increase in overeating during the pandemic among those who experienced more stressors due to the pandemic. The study result showed that not only family health, including resources, but also higher education, are significant protective factors against overeating. However, other aspects of family health were not shown to be associated with overeating besides family health resources.

## Figures and Tables

**Table 1:** Descriptive statistics, *N* = 443.

Variable	Mean (SD)
Female (%)	38.15
Age	37.27 (10.57)
Married or Cohabitating (%)	65.79
Bachelor’s Degree or Higher (%)	74.04
White/Caucasian (%)	71.33
Overeating Current	15.51 (11.24)
Overeating Before	14.72 (11.11)
COVID-19 Stressors	1.31 (1.10)

**Table 2:** Multiple linear regression of the effects of COVID-19 stressors and family health on overeating during the COVID-19 pandemic, *N* = 443.

Variable	Coef.
COVID-19 Stressors	0.63 *
Family Health (General)	0.15
Family Health Resources—full subscale	−1.09 ***
**Controls**	
Overeating before COVID-19	0.83 ***
Female	0.87 *
Age	0.03
Married or Cohabitating	0.26
Bachelor’s Degree or Higher	−0.96 *
White/Caucasian	−0.04

* *p* < 0.05. *** *p* < 0.001.

## Data Availability

Data are not publicly available due to IRB protocols for the study.
